# Experimental design of response surface methodology used for utilisation of palm kernel cake as solid substrate for optimised production of fungal mannanase

**DOI:** 10.1080/21501203.2016.1229697

**Published:** 2016-09-12

**Authors:** Saroj Ahirwar, Hemant Soni, Hemant Kumar Rawat, Bhanu Pratap Prajapati, Naveen Kango

**Affiliations:** Department of Microbiology, Dr. Harisingh Gour University, Sagar, India

**Keywords:** Agro-waste, fungi, palm kernel cake, β-mannanase, response surface methodology

## Abstract

The results obtained from this work strongly indicate that the solid state fermentation (SSF) system using the palm kernel cake (PKC) as a substrate is an economical method for the production of β-mannanase at extremely low operational cost based on the fact that PKC is one of the cheap and abundant agro-waste by-products of the palm oil industry. Under initial conditions, i.e. 2 mm particle size of PKC, the moisture ratio of 1:1 of PKC:moistening agent and pH 7, *Malbranchea cinnamomea* NFCCI 3724 produced 109 U/gram distribution of the substrate (gds). The production of β-mannanase was optimised by the statistical approach response surface methodology (RSM) using independent variables, namely initial moisture (12.5), pH (9.0) and solka floc (100 mg). Noticeably, six fold enhancement of β-mannanase production (599 U/gds) was obtained under statistically optimised conditions. HPLC results revealed that β-mannanase is an endo-active enzyme that generated manno-oligosaccharides with a degree of polymerisation (DP) of 3 and 4. Semi-native PAGE analysis revealed that *M. cinnamomea* produced three isoforms of mannanase. Selective production of oligosaccharide makes *M. cinnamomea* β-mannanase an attractive enzyme for use in food and nutraceutical industries.

## Introduction

The lignocellulosic biomass is composed of lignin, cellulose and hemicellulose. From the industrial point of view, economical and efficient conversion of these polymers, especially cellulose and hemicellulose, to their constituent monomer is very important (Moreira and Filho 2008). Hemicellulose is the second most abundant heteropolymer composed of mainly pentoses (xylose, arabinose), hexoses (mannose, glucose, galactose) and sugar acids (Chauhan et al. 2012). Mannan is the second major hemicellulose after xylan that occurs as a hemicellulose fraction of soft and hard wood. Endo-1, 4-β-D-mannanase (EC 3.2.1.78) is a hemicellulolytic enzyme that randomly hydrolyses 1,4-β-D-mannopyranosyl linkages within the main chains of mannans and heteropolysaccharides such as galactomannan, glucomannan and galactoglucomannan. Other accessory enzymes involved in mannan breakdown include β-mannosidase, β-glucosidase and α-galactosidase that liberate mannose, glucose and galactose from mannan (Dhawan and Kaur 2007; Soni and Kango 2013). Owing to the beneficial effects of pretreatment, mannanases have been widely used in various industries like food and nutraceutical industries (production of prebiotics), paper and pulp industry (enzymatic bleaching), detergent industry, poultry feed industry (feed upgradation) etc. (Dhawan and Kaur 2007; Soni and Kango 2013). Large amounts of agro-industrial lignocellulosic waste are generated and accumulated from various industrial activities that cause environmental pollution. Utilisation of this solid industrial waste, for producing value-added commodities and its disposal, can be mediated by microbial bioprocesses. Among these, SSF is a suitable method for the utilisation of agro-industrial by-products in the production of useful products like enzymes, organic acids, sugars etc. Enzyme-based bioconversion technologies are fastly emerging as an amicable solution for solid waste disposal (Pandey 2003).

The palm kernel cake is left-over as a by-product after the oil extraction process of the palm oil industry and contains a large number of non-starch polysaccharides with mannan (78%) as the major component (Dusterhoft et al. 1992; Keng et al. 2009). Therefore, it can serve as a readily available and low-cost substrate for commercial exploitation for mannanase production. The average cost of PKC has been estimated to be about 0.093/kg in 2015 (MPOB, 2015; Azman et al. 2016). Abdeshanian et al. (2010) and Soni et al. (2015) used PKC as a solid substrate in SSF for production of β-mannanase using mesophilic fungi *Aspergillus* sp. & *A. terreus*, respectively. Sanghvi et al. (2010) used another agricultural waste, wheat straw for production of thermostable xylanase using *Trichoderma harzianum.*

Thermophilic fungi are known to be excellent source of thermostable enzymes. However, only a few thermostable β-mannanases have been characterised from these (Puchart et al. 2004; Yang et al. 2015). Keeping this point in mind we evaluated a thermophilic fungus, *M. cinnamomea* NFCCI 3724 for optimised production of β-mannanase on PKC using the response surface methodology approach.

Environmental factors such as temperature, moisture content of the substrate and carbon supplements significantly affect SSF process, which in turn influences the product yield (Mohamad et al. 2011; Sadaf and Khare 2014; Soni et al. 2016). RSM is the collection of statistical techniques for experiment design, model development, evaluation factors and optimum conditions search (Neter et al. 1996). To enhance the production of β-mannanase by thermophilic *M. cinnamomea* NFCCI 3724 rotatable central composite design (RCCD) and experimental factorial design can be employed to optimise the medium conditions for enzyme production and perform a limited number of experiments.

Optimisation of β-mannanase production using copra meal as a solid substrate through RSM approach was successfully demonstrated by Ahirwar et al. (2016). To our knowledge, no data on β-mannanase production by thermophilic *M. cinnamomea* using PKC as the solid substrate in SSF is reported. In this work, statistical experimental design produced by RCCD was applied to study the combined effect of three important independent variables namely, pH, moisture and carbon supplement on β-mannanase production by *M. cinnamomea* NFCCI 3724. Mannose-free manno-oligosaccharides (MOS) are desirable as prebiotics in nutraceutical industries. Recently, Chauhan et al. (2014) and Dhawan et al. (2015) have demonstrated the role of MOS as prebiotic in supporting growth of probiotics (*Lactobacillus* and *Bifidobacterium*) and inhibiting the growth of pathogen (*Salmonella enterica*). The focus of this study is the optimised production of β-mannanase on a solid substrate (PKC), a by-product of palm oil industry, and enzymatic hydrolysis of mannan (locust bean gum and konjac gum) for the generation of MOS.

## Materials and methods

### Chemicals

Mannobiose (M_2_), mannotriose (M_3_) and mannotetraose (M_4_) were purchased from Megazyme (Bray, Ireland). Locust bean gum (LBG), solka floc, glucose, mannose, guar gum, *p*-nitrophenyl-substrates, *p*-nitrophenol and other chemicals were procured from Sigma-Aldrich, USA. Yeast extract, peptone, urea and ammonium sulphate were procured from Hi-Media, India. The palm kernel cake was purchased from M/s Meh Impex, Chennai, India. Food grade konjac gum (glucomannan) was obtained from New Foods, Illinois, USA. Fenugreek seed meal (seed meal of *Trigonella foenum-graecum*), wheat bran and wheat straw were purchased from local market.

### Fungal strain

The fungus *Malbranchea cinnamomea* was isolated from district Sagar, Madhya Pradesh, India, during a survey of thermophilic fungi from litter in the year 2013. It was identified on the basis of colony morphologies and structural characteristics as observed under the light and scanning electron microscopy and deposited with National culture collection of India, Pune, with accession number 3724. The fungal characteristics were described and identified based on the description given by Cooney and Emerson (1964). It was maintained on yeast extract phosphate-soluble starch agar (YpSs) slants at 4ºC and sub-cultured after every 30 days.

### Inoculum preparation and solid state fermentation

*M. cinnamomea* NFCCI 3724 culture grown on YpSs at 45°C for 7 days was used for the preparation of spore suspension. Spores were harvested using 5 ml sterile 0.01% (w/v) Tween 80 with the aid of wire loop (Soni et al. 2015). Various substrates like wheat bran, wheat straw, palm kernel cake, and fenugreek seed meal were used for β-mannanase production. Different sieves were used for obtaining 0.5, 1.0, 2.0 particle size of the palm kernel cake. Fermentation was carried out in Erlenmeyer flask (250 ml) with 5 g substrate with a 1:1 ratio of moisture content (distilled water) and yeast extract, peptone, urea and ammonium sulphate were used as nitrogen supplements (1% w/v). Flasks were then autoclaved at 121°C for 30 minutes and inoculated with 1 ml of spore suspension (2×10^6^ spores/ml) of *M. cinnamomea* NFCCI 3724 and incubated at 45°C for 7 days.

### Enzyme extraction

After incubation, 50 ml Na-citrate buffer (pH 5.0) was added to each flask and kept for shaking at 150 rpm for 1 h at 4°C temperature. For extraction, entire content of flask was squeezed through a muslin cloth and the extract was centrifuged at 9000 g for 15 min at 4°C and cell-free clear supernatant was used as source of mannanase.

## Enzyme assays

### β-Mannanase

β-Mannanase activity was measured using locust bean gum (0.5% w/v) as a substrate. LBG was dissolved in 0.05 M Na-citrate buffer (pH 5.0) by stirring constantly for 1 h at 60°C and clear solution was obtained by centrifugation at 10,000 g for 10 min. 100 μl enzyme sample was added to 900 μl substrate and incubated at 50°C for 10 min. The reaction was stopped by adding 1.5 ml dinitrosalicylic acid (DNS) reagent and boiling at 100°C for 5 min. Reducing sugar was measured at 540 nm against the blank (Miller 1959). One unit (1U) of enzyme activity was defined as the amount of enzyme required to produce 1μmol of reducing sugar per minute under experimental conditions.

### Assay of accessory enzymes (β-mannosidase, α-galactosidase and β-glucosidase)

900 µl of P-nitrophenyl-β-D-mannopyranoside (2 mM in 0.05 M Na-citrate buffer, pH 5.0) was incubated with 100 µl enzyme sample at 50ºC for 10 min. The reaction was stopped by adding 0.5 ml of 1 M Na_2_CO_3_ and absorbance of *p*-nitrophenol released was determined at 400 nm (Maijala et al. 2012). One unit (1U) of β-mannosidase was defined as the amount of enzyme that liberated 1 µmol *p*-nitrophenol in 1 min. α-galactosidase and β-glucosidase were measured in a similar manner except that *p*-nitrophenyl-α-D-galactopyranoside (2 mM) and *p*-nitrophenyl-β-D-glucopyranoside (1 mM) were used as a substrate, respectively.

### Optimisation studies by the response surface methodology (RSM) and validation of experimental design

Moisture content, pH and carbon supplement are important factors affecting the production of enzyme in solid state fermentation (Mohamad et al. 2011; Sadaf and Khare 2014). In this study three selected factors pH (A), moisture content (B) and solka floc as carbon supplement (C) were selected to find their optimum values for β-mannanase production by *M. cinnamomea* NFCCI 3724 using rotatable central composite design (RCCD) of RSM. A total of 20 set of experiments including six centre points were conducted along with different combinations of selected parameters.

The data obtained from RSM was subjected to analysis of variance (ANOVA) for analysis of regression coefficient, prediction equations and case statistics. Analysis of data was performed using Design-Expert software (Version 9.0). The experimental results of RSM were fitted using the second order polynomial Equation (1):

(1) $$\begin{array}{c} Y=\beta 0+\sum_{i}\beta iXi+\sum_{ii}\beta iiXi2+\sum_{ij}\beta ijXiXj \end{array}$$

where Y is the predicted response, Xi, Xj are independent variables, β0 is the intercept term, βi is the linear coefficient, βii is the quadratic coefficient, and βij is the interaction coefficient. The statistical model was validated with respect to all variables within the design space. A random set of six experimental optimised combinations was used to study the β-mannanase production under SSF using PKC as a substrate.

### Detection of mannanase enzyme activity by semi-native SDS-PAGE

For detecting the activity of mannanase, the SDS-PAGE (12%) gel containing 2 ml of substrate konjac gum (0.5% w/v) was subject to electrophoresis. After electrophoresis the gel was washed for 10 min in distilled water then washed with 25% (v/v) propane in Na-citrate buffer (pH 5.0) followed by washing with double distilled water. The gel was incubated at 50°C for 5 min under shaking in 50 mM Na-citrate buffer (pH 5.0) solution and stained with 0.1% Congo red solution for 20 min. Finally, the gel was destained with NaCl (1% w/v in distilled water) for 1 h (Rajulu et al. 2011).

### Hydrolysis experiments and end product analysis

Total protein of the filtrate obtained after enzyme extraction was precipitated by adding double volume of chilled ethanol under constant stirring at 4°C. The mixture was then kept at 20°C overnight to allow complete precipitation. The precipitate was collected by centrifugation at 8000 g for 15 min. Precipitate thus obtained was re-suspended in Na-citrate buffer pH 5.0 and dialysed using dialysis membrane (10,000 molecular weight cut-off, Hi-Media, India) against the same buffer at 4°C overnight (Atac et al. 1993). The dialysed sample was used for hydrolysis of LBG and konjac gum. Hydrolysis of two mannan types, galactomannan (LBG) and glucomannan (konjac gum) (0.5% w/v in Na-citrate buffer, pH-5.0) was conducted by incubating equal volumes of substrate and enzyme (25 U/ml) (1:1) at 50°C with constant shaking. Samples were withdrawn at regular intervals of 10 h and boiled to stop the reaction. Sample was filtered through membrane filter (0.45 µm) and the filtrate was analysed by HPLC (Waters, USA) using Sugar Pak column, RI detector 2414 and injection valve of 20 µl (Soni et al. 2015). Water (HPLC Grade) was used as mobile phase at flow rate of 0.5 ml/min and column temperature was 90°C. Analysis was done by Empower 2 Buildsoftware 2154.

## Results and discussion

### Solid state fermentation for the production of mannanase by M. cinnamomea

**Figure 1.**
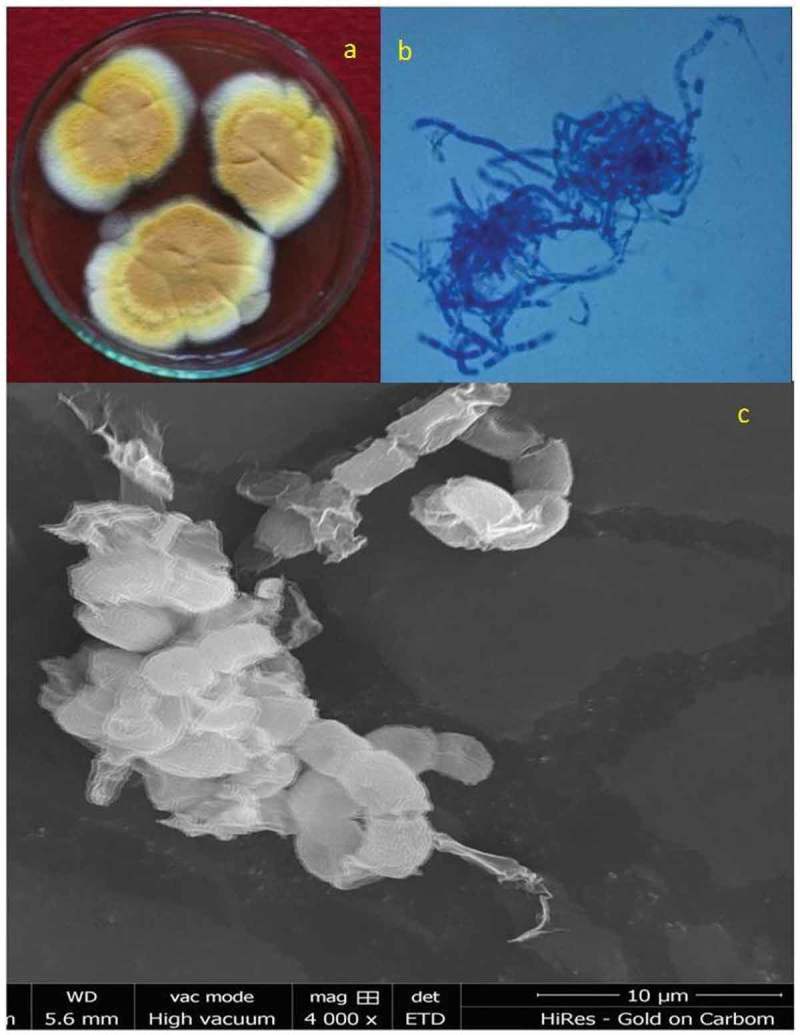
Locally isolated *M. cinnamomea* NFCCI 3724 under scanning electron microscopy showing structural morphologies.

The fungus was identified based on the morphological details as observed under the light and scanning electron microscopes (SEM). Growth on YpSs showed that the initial white mycelia turned yellow upon maturation (Figure 1). Based on these characteristics, fungus was identified to be *M. cinnamomea* (Cooney and Emerson 1964).

**Figure 2.**
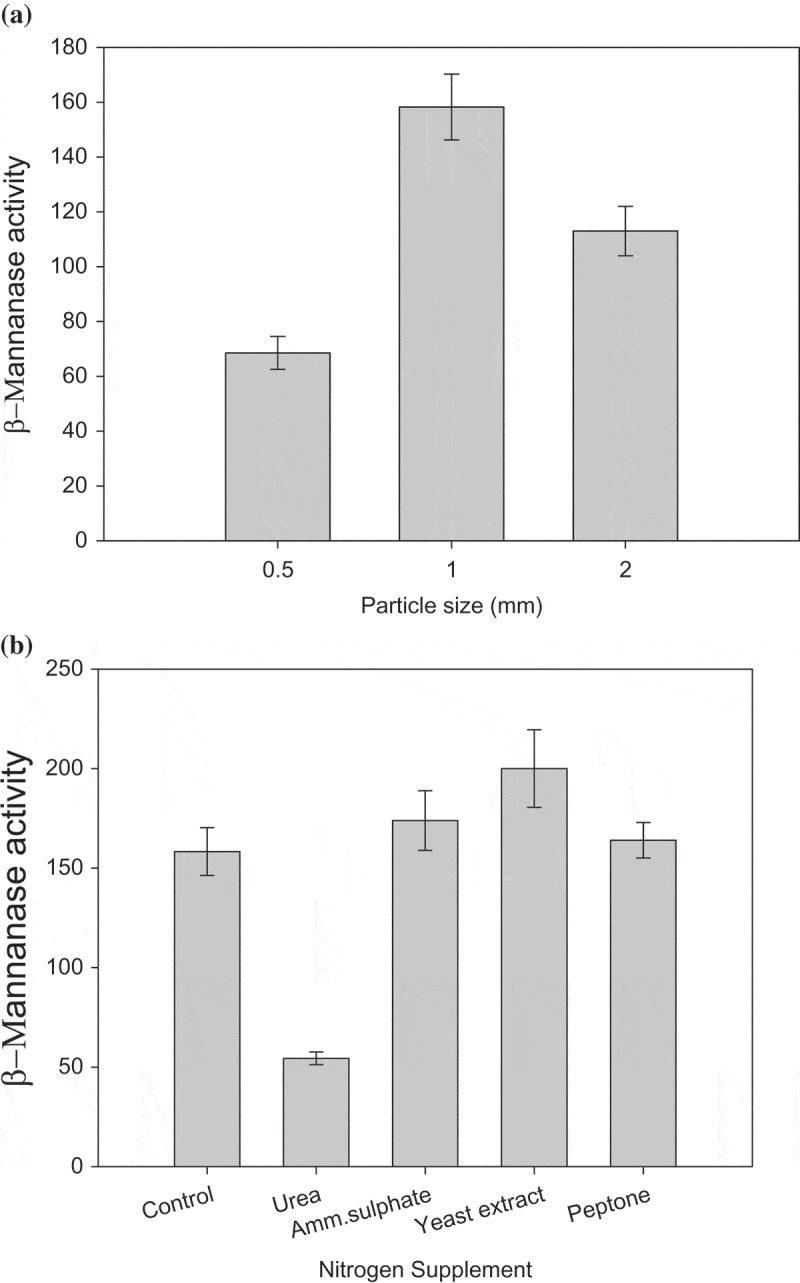
Effect of various process parameters on β-mannanase by *M. cinnamomea* NFCCI 3724 on PKC as solid substrate in SSF; (a) Effect of particle size of PKC; (b) Effect of nitrogen supplementation (Culture conditions: Incubation period 8 days, Incubation temperature 45°C).

Among the four substrates examined for production of β-mannanase in SSF, PKC was observed to be the best supporting 109 U/gds β-mannanase (Table 1). Henceforth PKC was used for mannanase production in further studies. Recent, reports suggest use of PKC in mannanase production by mesophilic fungi (Abdeshanian et al. 2010; Rashid et al. 2011; Soni et al. 2015). However, to date there has been no report of β-mannanase production on PKC by any thermophilic fungi. Optimisation of various parameters *viz*. particle size of the substrate and nitrogen supplementation was carried out. Among the particle sizes examined for β-mannanase production, particle size, 1 mm supported maximum yield 158 U/gds (Figure 2(a)). Sadaf and Khare (2014) have reported the substrate particle size of 0.6 mm suitable for xylanase production by another thermophilic mold, *Sporotrichum thermophile*. Among the nitrogen supplements examined, yeast extract supported maximum yield (200 U/gds) comparable to the unsupplemented control (158 U/gds). It is clearly evident from Figure 2(b) that urea adversely affected β-mannanase production. These results are well supported by the earlier reports on hemicellulase production where organic nitrogen sources, particularly, yeast extract has been found to enhance enzyme production (Gaffney et al. 2009) while urea supplementation repressed β-mannanase production (Betini et al. 2009).

**Table 1.** Production of mannan degrading enzymes on various low value substrates. Data points indicate the means of triplicate values ± SD.

Substrate	β-Mannanase U/gds	β-Mannosidase U/gds	α-Galactosidase U/gds	β-Glucosidase U/gds
PKC	109 ± 3.2	0.2 ± 1.7	0.4 ± 1.5	0.3 ± 0.5
Wheat bran	16 ± 2.5	BDL	BDL	BDL
Fenugreek gum	6 ± 2.3	BDL	BDL	BDL
Wheat straw	8 ± 1.8	2.3 ± 0.2	1 ± 0.2	1.6 ± 0.1

BDL: Below detection level

### Optimisation studies by the response surface methodology (RSM)

In this investigation, RSM was employed to study the interactions among the three independent variables (pH, moisture and solka floc as carbon supplement) to determine their optimal levels. RSM involving a RCCD was adopted to optimise the selected parameters for β-mannanase production by *M. cinnamomea* NFCCI 3724. A set of 20 experiments including six center points was carried out and each numeric factor was varied over five levels (-α, -1, 0, +1, +α). The full experimental plan with respect to their actual and coded values is listed in Table 2. The response values (Y = mannanase activity) in each trial were the average of the triplicates. ANOVA was applied for analysis of regression coefficient, prediction equations and case statistics. The experimental results of RSM were fitted using the following second order polynomial Equation (1). In this study, the independent variables were coded as pH (A), moisture (B) and solka floc as carbon supplement (C). The second-order polynomial model for the mannanase activity was expressed by Equation (2).

**Table 2.** Experimental rotatable central composite design (RCCD) matrix for different variables with actual β-mannanase production. Data points indicate the means of triplicate values ± SD.

Std. Run	Level	A:pH	B:Moisture (ml)	C:Solka floc (mg)	β-Mannanase Activity (U/gds)	
	-α	3.9	1.2	19	Predicted	Actual
	−1	6	6	60		
	0	9	13	120		
	+1	12	20	180		
	+α	14	24.7	220		
1		6	6	60	390.05	385 ± 12
2		12	6	60	322.65	330 ± 18
3		6	20	60	325.93	338 ± 10
4		12	20	60	308.09	298 ± 17
5		6	6	180	233.45	240 ± 8
6		12	6	180	300.47	284 ± 5
7		6	20	180	211.89	201 ± 4.5
8		12	20	180	328.49	330 ± 19
9		3.9	13	120	213.80	210 ± 7
10		14	13	120	255.17	264 ± 22
11		9	1.2	120	335.16	338 ± 13
12		9	24.7	120	304.80	307 ± 11
13		9	13	19	454.75	450 ± 6
14		9	13	220	340.22	350 ± 8
15		9	13	120	559.47	576 ± 24
16		9	13	120	559.47	570 ± 21
17		9	13	120	559.47	558 ± 28
18		9	13	120	559.47	557 ± 19
19		9	13	120	559.47	545 ± 17
20		9	13	120	559.47	550 ± 14

(2) $$\begin{array}{l} \text{Y}\left(\text{Coded}\right)=\text{+559}.\text{48+12}.\text{30*A-9}.\text{03*B}\\ -34.05*\text{C}+12.39*\text{AB}\\ \text{+33}.\text{61}+10.64*\text{BC}-114.90\\ {}*{\text{A}}^{2}-84.67*{\text{B}}^{2}-57.27*{\text{C}}^{2}\\ -57.27*{\text{C}}^{2} \end{array}$$

**Figure 3.**
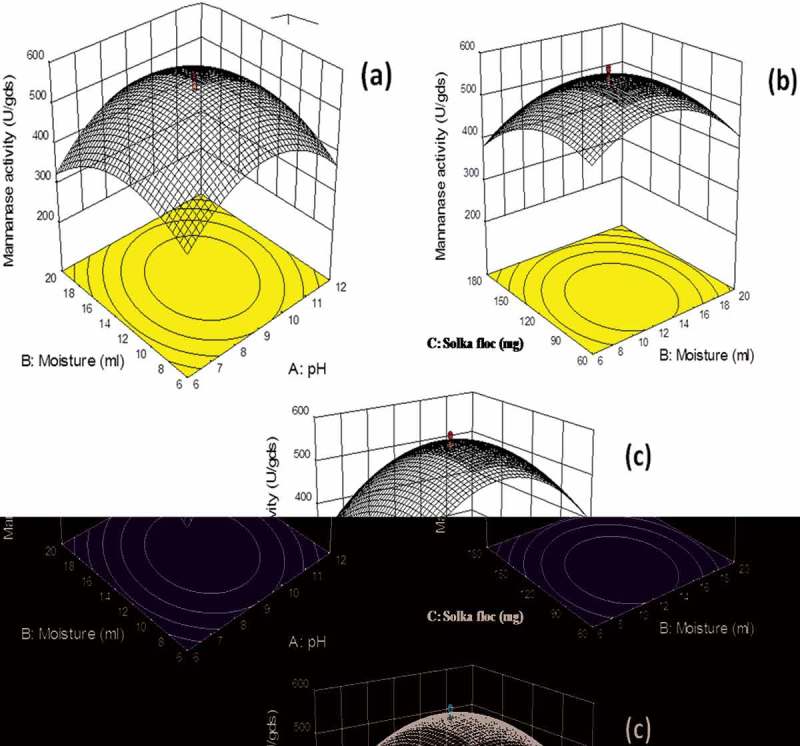
Three dimensional response surface curves for mannanase production (U/gds) by *M. cinnamomea* NFCCI 3724; (a) Effect of moisture and pH on β-mannanase production; (b) Effect of solka floc and moisture content on β-mannanase production (c) Effect of solka floc and pH on β-mannanase production (Culture conditions: Incubation period 8 days, Incubation temperature 45°C).

**Figure 4.**
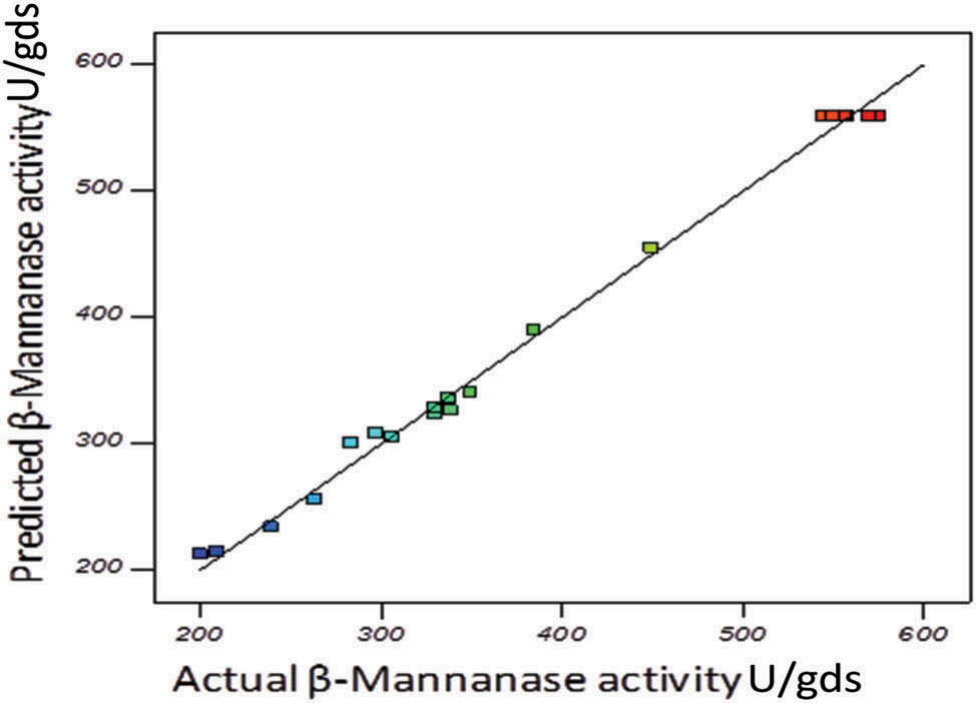
Predicted vs. actual response plot of the quadratic model used for β-mannanase production (U/gds) by *M. cinnamomea* NFCCI 3724.

The adequacy of the model was checked by using ANOVA, which was tested through Fisher’s statistical analysis and the results are shown in Table 3. The model *F*-value 210.02 implied that the model was significant and there is only 0.01% chance that “model *F*-value” could occur due to noise. The ‘Lack of Fit’ of *F*-value was 1.46, which indicated that ‘Lack of Fit’ was also not significant relative to the pure error. There was 34.46% chance that a “Lack of Fit *F*-value” could occur due to noise. The “Pred R-Squared” of 0.9702 is in reasonable agreement with the “Adj R-Squared” of 0.9900. Here, the coefficient of variation (CV) indicated that degree of precision with which the experiments were compared. The higher reliability of the experiment was usually indicated by lower value of CV. In the present study, the CV of the model was 3.39% proving the higher precision as well as reliability concerning the experiments. All the statistic results of the model showed that accuracy and general applicability of the second-order polynomial equations, and they were adequate to describe the responses of experiments. The values of ‘Prob>*F*’ were employed to check the signiﬁcance of each model term which, in turn were of vital importance for the understanding of the pattern of the mutual interactions between the experiment variables. Values of ‘Prob>*F*’ less than 0.0500 indicated model terms were the most significant. Table 2 showed that linear term of the independent variables including pH and moisture content a significant effect on the mannanase activity of fungus. The quadratic terms of the three variables and the interaction between AB, AC and BC also had significant effects. Figure 3(a,b,c) showed the response surface contour plots for the β-mannanase production generated by the predicted model, respectively. It was observed from the statistics that the β-mannanase production has increased significantly until the moisture content reached up to 13 ml, and then it decreased (Std. Run no. 3, 4, 7 and 8). The effect of pH on the β-mannanase production was also sensitive in the designed range, which could also be explained by the *p*-value (<0.0001) in Table 3. The β-mannanase production had almost constant increase in the designed range of solka floc from 60 to 120 mg. Predicted vs. actual plot (Figure 4) represented a high degree of similarity that was observed between the predicted and experimental values. From the diagnostic plots it can be concluded that the model satisfied the assumptions of the analysis of variance and also reflected the accuracy and applicability of RSM to optimise the process for β-mannanase production.

**Table 3.** Analysis of variance (ANOVA) of the quadratic model of rotatable central composite design (RCCD).

Source	Sum of Squares	DF	Mean Square	*F*-value	*p*-value (Prob > *F*)	
Model	3.212E+005	9	35685.22	210.02	< 0.0001	Significant
A-pH	2065.52	1	2065.52	12.16	0.0059	
B-Moisture	1112.71	1	1112.71	6.55	0.0284	
C-Carbon	15835.59	1	15835.59	93.20	< 0.0001	
AB	1228.52	1	1228.52	7.23	0.0227	
AC	9035.91	1	9035.91	53.18	< 0.0001	
BC	906.04	1	906.04	5.33	0.0436	
*A*^2^	1.903E+005	1	1.903E+005	1119.73	< 0.0001	
*B*^2^	1.033E+005	1	1.033E+005	608.06	< 0.0001	
*C*^2^	47269.12	1	47269.12	278.19	< 0.0001	
Residual	1699.16	10	169.92			
Lack of Fit	1007.83	5	201.57	1.46	0.3446	Not significant
Pure Error	691.33	5	138.27			
Cor Total	3.229E+005	19				

*R* ^2^ = 0.9947; Pred *R* ^2^ = 0.9702; Adj *R* ^2^ = 0.9900

### Validation of the model

To validate the suggested mathematical model, an additional experiment was conducted under the predicted optimal conditions (pH 9.0, moisture content 12.5 ml and 100 mg solka floc). The experimental β-mannanase production, 599 U/gds under optimised condition was higher than the predicted β-mannanase production (564 U/gds). Therefore, the model developed was reliable for predicting the optimal conditions for variables influencing the β-mannanase production. Production of β-mannanase by RSM increased six-fold than unoptimised (Table 4). Amount of other associated hemicellulases, β-mannosidase, β-glucosidase and α-galactosidase were 0.28 U/gds, 0.31 U/gds and 0.43 U/gds, respectively. The cocktail of these mannan hydrolysing enzymes is reported to be useful in achieving complete hydrolysis of mannan by hetero and homosynergistic action (Malgas et al. 2015).

**Table 4.** Analysis of β-mannanase production by various approaches.

Approach	β-Mannanase production (U/gds)	Fold increase
Unoptimised	109	1
One variable at a time	200	~2
Statistically optimised (RSM)	599	~6

### Detection of mannanase enzyme activity by semi-native SDS-PAGE

**Figure 5.**
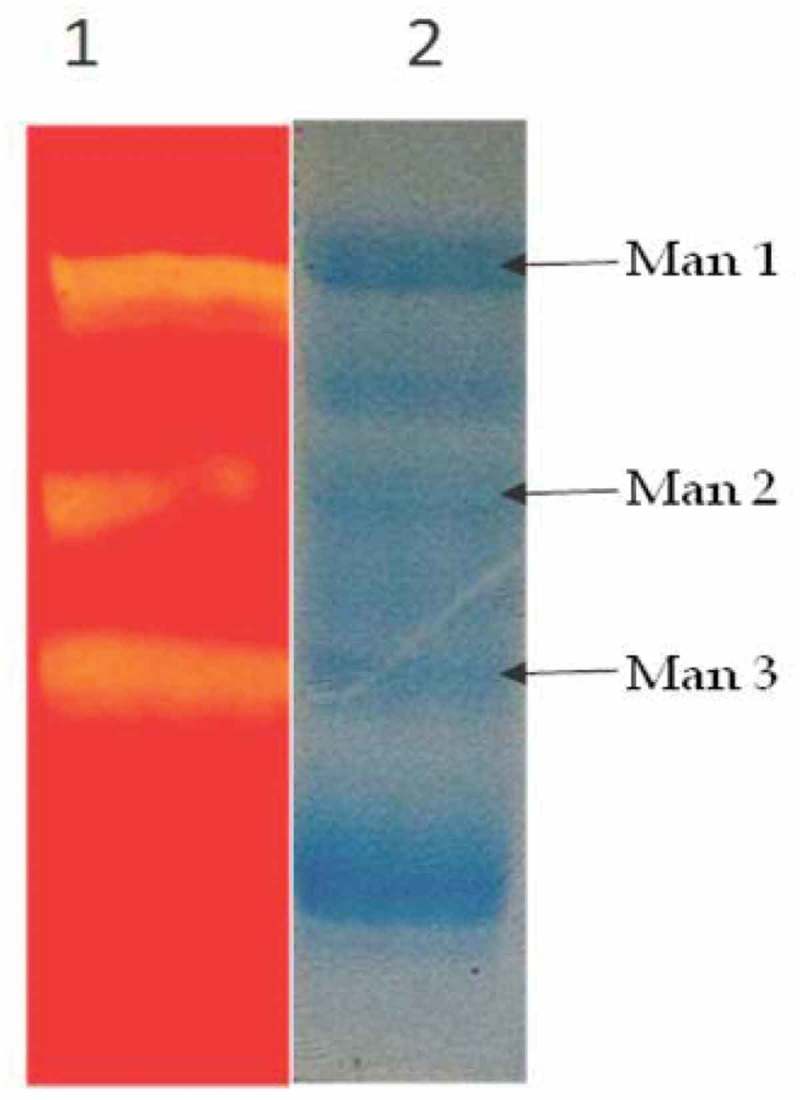
Zymogram analysis crude enzyme *Lane 1* zymogram for mannanase *Lane 2* SDS-PAGE profile of crude enzyme.

A semi-native SDS-PAGE analysis of mannanase preparation obtained from solid state cultures on PKC revealed that *M. cinnamomea* NFCCI 3724 produced different isoforms of mannanase acting on konjac gum. *M. cinnamomea* NFCCI 3724 produced three isoforms of mannanase (Figure 5).

### Mannan hydrolysis and manno-oligosaccharide generation

**Figure 6.**
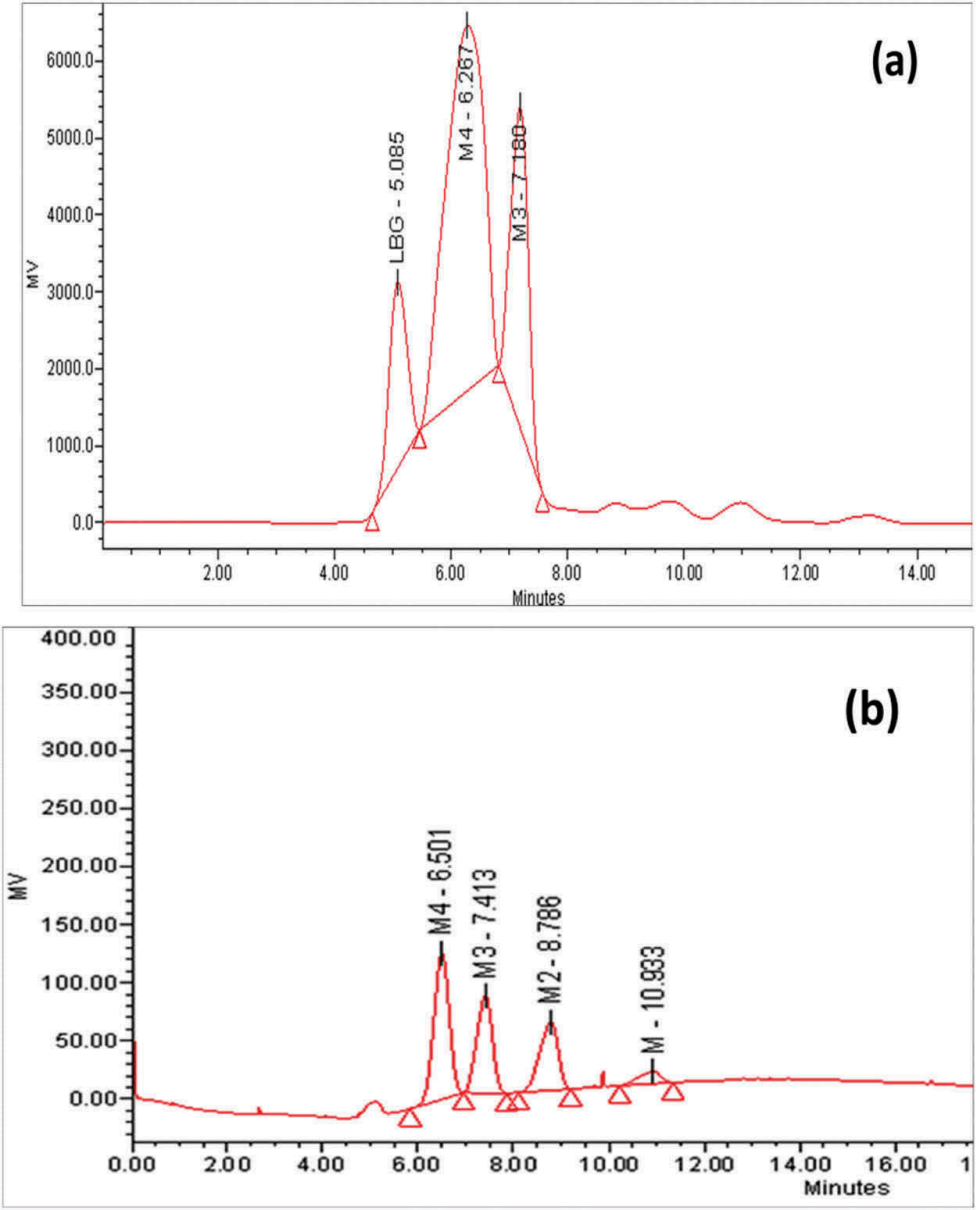
HPLC analysis of mannan hydrolysis: Equal volumes (1 ml each) of substrate (0.5% w/v) and mannanase (36 U/mg) at 50 °C under constant shaking. a) Hydrolysis of LBG; b) Standards, mannose (M), mannobiose (M_2_), mannotriose (M_3_), mannotetraose (M_4_).

Partially purified *M. cinnamomea* β-mannanase was applied for mannan (LBG) hydrolysis and products were analysed by HPLC. The results obtained after hydrolysis are shown in Figure 6.

β-Mannanase hydrolysed 40% (degree of polymerisation of 3) and 58% (degree of polymerisation of 4) w/v of galactomannan (LBG). The major products liberated from LBG were oligosaccharide with degree of polymerisation of 3 & 4. This result is comparable with Hakamada et al. (2014) and Blibech et al. (2011). Recently, β-mannanase from thermophilic fungus *Neosartorya fischeri* P1 (Yang et al. 2015) significantly hydrolysed both konjac gum and LBG and generated mannose, M_2_, M_3_, M_4_, M_5_ and M_6_ with very low amount of M_4_ while in this study β-mannanase produced M_3_ and M_4_ from LBG. Lack of mannose formation from LBG suggested that *M. cinnamomea* produced highly endo active β-mannanase without any associated β-mannosidase activity, a property important in generation of MOS.

## Conclusions

Our findings suggest that among the various substrates tested in this study, PKC by far was the best substrate for the economical production of thermostable β-mannanase by *M. cinnamomea* NFCCI 3724. Yeast extract was the best nitrogen supplement among the other sources tested for higher yield of β-mannanase. About six-fold enhancement in β-mannanase production (599 U/gds) was achieved through optimisation of parameters (pH, moisture content and solka floc as carbon supplement) using statistical experiment design. Mannanase effectively hydrolysed various mannans and produced MOS (mannotriose and mannotetraose) selectively. This indicated its applicability in prebiotic generation from gums. The study also provided an approach for utilisation and management of agro-industry solid by-products which causes pollution and its use in economic enzyme production. After the reduction in mannan content of the palm kernel cake it may also be used as feed for monogastric animals.
